# Absent without leave; a neuroenergetic theory of mind wandering

**DOI:** 10.3389/fpsyg.2013.00373

**Published:** 2013-07-01

**Authors:** Peter R. Killeen

**Affiliations:** Department of Psychology, Arizona State UniversityTempe, AZ, USA

**Keywords:** ADHD, attentional lapses, attractors, Markov model, response times, Wald distribution

## Abstract

Absent minded people are not under the control of task-relevant stimuli. According to the Neuroenergetics Theory of attention (NeT), this lack of control is often due to fatigue of the relevant processing units in the brain caused by insufficient resupply of the neuron's preferred fuel, lactate, from nearby astrocytes. A simple drift model of information processing accounts for response-time statistics in a paradigm often used to study inattention, the Sustained Attention to Response Task (SART). It is suggested that errors and slowing in this fast-paced, response-engaging task may have little to due with inattention. Slower-paced and less response-demanding tasks give greater license for inattention—aka absent-mindedness, mind-wandering. The basic NeT is therefore extended with an ancillary model of attentional drift and recapture. This Markov model, called NEMA, assumes probability λ of lapses of attention from 1 s to the next, and probability α of drifting back to the attentional state. These parameters measure the strength of attraction back to the task (α), or away to competing mental states or action patterns (λ); their proportion determines the probability of the individual being inattentive at any point in time over the long run. Their values are affected by the fatigue of the brain units they traffic between. The deployment of the model is demonstrated with a data set involving paced responding.

## Introduction

The enduring stereotype of the absent-minded professor speaks both to the abstraction of his profession and to its diffuse impact on the common weal. The absent-minded neurosurgeon and the absent-minded airplane pilot are sooner terminated, by ethical panels or by mountain ranges. In all cases absentmindedness is seen as a fault, one whose gravity depends on the importance and delicacy of the task left unattended. Fantasy, daydreams and night-dreams are mind wandering at its purest, typically let manifest in secure environments of easy chair or bed. It is only when the mind is absent from a high-priority task without leave that its owner may incur sanctions. Why does the mind go AWOL?

In their seminal paper *The Restless Mind*, Smallwood and Schooler (2006), argue that mind wandering is due to the hijacking of attention away from the primary task by an alternative goal that becomes activated without meta-awareness. Once interrupted, attention to the primary task is delayed or forgotten. Much mischief may issue from restless minds, ranging from neglect of desired or essential actions, to inappropriate displacement of those actions by other actions—slips. Slips may be verbal (Freud, 1966), musical (Palmer and Van De Sande, 1993), or non-acoustic actions (Norman, 1981). They are commonplace (Cheyne et al., 2006), more so under conditions of fatigue or emotion, when more common behavioral trajectories intersect with less common ones (Heckhausen and Beckmann, 1990), and in special populations (Robertson et al., 1997). Hypnosis subverts meta-awareness, giving the hypnotist control over the direction in which the mind will wander, and the actions that will ensue (Hilgard, 1992; Woody et al., 1992; Killeen and Nash, 2003).

This article addresses, not where the mind goes when it is absent, but why it leaves. In particular, it proposes that in many cases, it is not that the mind is attracted toward other thoughts and actions, but rather that it flees from a difficult task. The task may be difficult because of fatigue or boredom—not in general terms, but in particular ones. One population that is especially susceptible to absent-mindedness is that of individuals with attention deficit disorder (ADHD, Inattentive and Combined subtypes). A recent theoretical treatment of that condition is introduced and applied to the general case of absent-mindedness.

## A neuroenergetics theory of attention (NeT)

Todd and Botteron (2001) hypothesized that ADHD might be due, not to a dopaminergic disorder, the contemporary and still regnant hypothesis, but rather to catecholamine-mediated hypofunctionality of astrocyte glucose and glycogen metabolism. Glial cells, which the brain contains in numbers about equal to that of neurons, surround the neurons. Some form the myelin sheath that makes neuroconduction much faster and more efficient. Others, the astrocytes, take up glucose from the capillaries and convert it to glycogen and lactate. Upon stimulation, the astrocytes release the lactate into the extracellular space, which the neurons can take up, and in turn convert to ATP, which fuels the many processes involved in signaling and reestablishment of gradients. Todd and Botteron hypothesized that reduced catecholaminergic input leads to a decrease in astrocyte-mediated neuronal energy metabolism and impaired frontal-cortex function in ADHD. Russell et al. (2006) took that hypothesis a step further, and addressed a specific aspect of the clinical presentation, moment-to-moment fluctuations in task performance that are often manifest in behavior of clinical populations, and refined and extended the biochemical bases underlying the hypoenergetic thesis. They hypothesized that in the case of ADHD both the oligiodendrocytes—the white matter of the brain that insulates the neurons—and also the astrocyte-neuron lactate shuttle that transported the energy from astrocyte to neuron, were compromised.

The latest step in this investigation has been the refinement of the energetic hypothesis based on our quickly accruing understanding of the neurochemical bases of this complex system (Bélanger et al., 2011; Jakoby et al., 2012), and the development of a mathematical model that carries the energetic hypothesis into direct contact with behavioral data (Killeen et al., 2013).

### Fuel and fatigue

Paying attention is an effortful process (Kahneman, 1973; Sarter et al., 2006). The effort involves the creation and transmission of action potentials, postsynaptic potentials, and the resetting of ion gradients, which are all energy-intense, yet essential for information transmission (Attwell and Gibb, 2005; Strelnikov, 2010; Howarth et al., 2012). The energy is initially provided by mitochondrial respiration, and subsequently by glial (astrocyte) processes (Mangia et al., 2003). The latter are triggered by the glutamate released by neurons, which is taken up by the astrocytes, powered by sodium influx. The ATP used to re-establish the sodium gradient stimulates the conversion of glycogen (glycogenolysis), which restores the ATP and also generates lactate (Kasischke et al., 2004; Hyder et al., 2006). Astrocytes' recruitment of energy from their glycogen stores (Benarroch, 2010) is facilitated by noradrenergic stimulation of the astrocytes' β-adrenoceptors (Fillenz et al., 1999; Hertz et al., 2010). Lactate is transported to the interstitial space, where it is incorporated and used by neurons as their preferred energy source (Pellerin et al., 2007). Insufficient supplies of glucose or lactate impair the release of glutamate from presynaptic terminals (Magistretti, 2009). The basic fuel stock for these processes is glucose, whose uptake is ultimately reflected in the BOLD signals sensed by fMRI (Engstrom et al., 2013).

The energy transport system in the brain is much more complicated than indicated by the above summary (Cloutier et al., 2009). The key point is that the brain, comprising only 2% of the body's weight, utilizes 25% of total glucose production for perception, attention, and response generation (Zhang and Raichle, 2010). An insufficiency in any of the links in the supply chain will slow information processing. One of the key buffers for energy deployment, the glycogen stores of the astrocytes, takes hours to replete, and that process is inhibited by noradrenergic stimulation concomitant to neural activity. Functional units in the brain will deplete energetic resources in their neurons over the course of a dozen seconds, and will draw down resources from the astrocytes over the course of dozens of minutes. This fatigue of those units slows their responsiveness, and increases the difficulty in engaging, or reengaging them. According to this hypothesis, energetic insufficiency is the main cause of inattention, distractibility and mind wandering—all of which constitute an escape to less fatigued functional units.

### Response generation: the NeT

**Figure 1:**
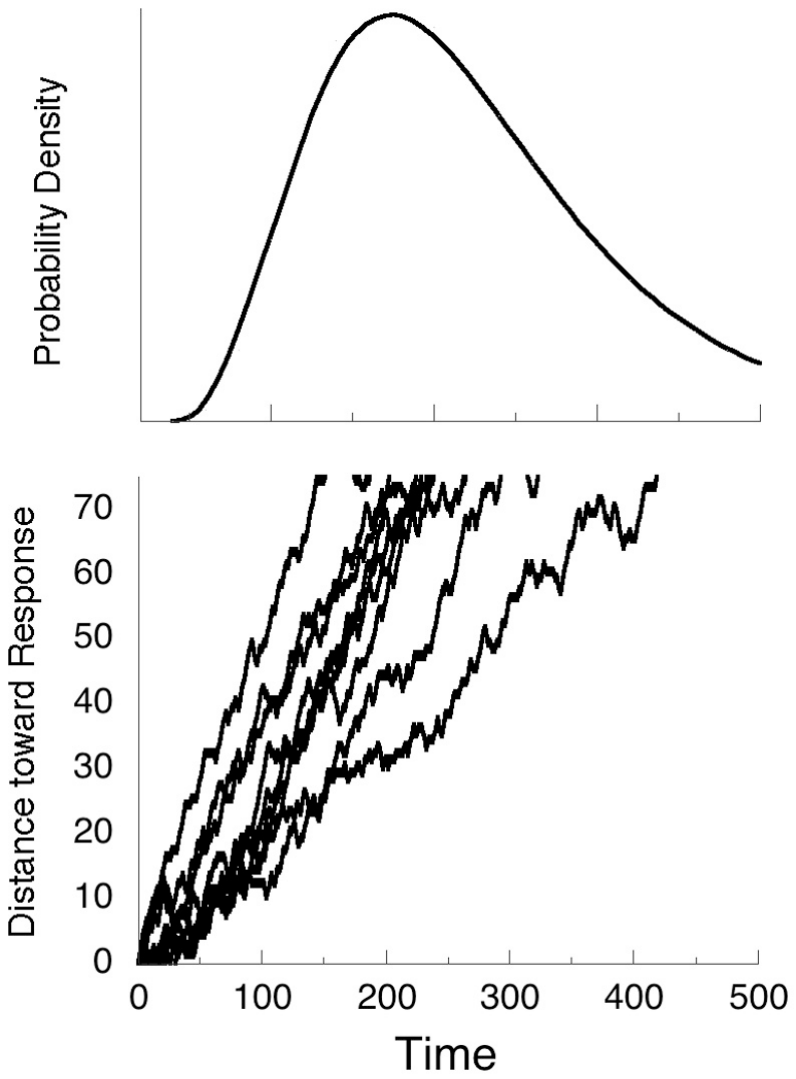
**Nine trajectories of random walks, with the probability of a step North twice the probability of a step South**. The distribution of times the trajectories cross the criterion *C* = 75 for the first time is given by the Wald density shown in the top panel.

The hundreds of thousands of neural events associated with a single response may be thought of as a tug of war between excitatory and inhibitory forces, with the drift toward the execution of the response a drunkards walk along the line. If a criterion distance of *C* = 75 units is set, the probability of a step in the positive direction is 2/3, and in the negative direction 1/3, and a step is made every millisecond, we get the trajectories shown in Figure 1. In some cases, the first hitting time at *C* = 75 is fast—100 ms; and in some cases slow—over 400 ms. If we redo the simulations thousands of times, and plot the proportion of times the criterion is crossed as a function of the total number of time-steps, the curve at the top results. It is the special case of the inverse Gaussian distribution (Chhikara and Folks, 1989) called the Wald distribution, given by:

(1) $$f(t)=\frac{c}{\sqrt{2\pi t^{3}}}e^{-\frac{{\left(c-vt\right)}^{2}}{2t}}\;t>0$$

*C* is the criterion, *t* time (here in ms), and *v* is the net velocity in steps per millisecond.

The mean of Eq. (1) is *m* = *C*/*v*, its standard deviation (*C*/*v*^3^)^1/2^, and its coefficient of variation is therefore (*Cv*)^−1/2^. The Wald is a skewed distribution with a coefficient of skewness equal to 3(*Cv*)^−1/2^. These statistics are heavily dependent on the speed of propagation: Halving the velocity *v* doubles the mean, and more than doubles the standard deviation. This explains why variability is more diagnostic of slowed neural processing speed than are means in various experimental and clinical conditions.

The primary assumption of this Neuroenergetics Theory of attention (NeT) is that focused attention to stimuli, especially to simple unvarying stimuli, fatigues the relevant functional units in the brain, and slows the processing speed, *v*. Different tasks require more or fewer computations (*C*), and these may differ among individuals and populations. In order to maintain a minimal response speed, individuals may sacrifice accuracy by decreasing the criterion for a response; this is often the case when task complexity is increased without increasing time allowed for the task. In the original development (Killeen et al., 2013) the velocity of neural propagation, *v*, was identified with the energy available for a response, *E*. That interpretation remains, but the equation is not necessary for present purposes, and so the parameter is kept closer to its origin.

Some experimental paradigms require the occasional inhibition of a response, either by presenting a rare non-target, or by presenting a supervening stop signal. It requires a finite time, *T*_stop_, to abort an initiated response. In the case of Figure 1, if *T*_stop_ is 150 ms, some fast trajectories will have completed before they can be aborted. These are often counted as errors of commission, or false positives.

Computation of these parameters is straightforward given the response-time distributions; but those are seldom given. Therefore, it is necessary to impute those parameters from known statistics of the distributions. Given the mean (μ) and standard deviation (σ) of the data, then:

(2) $$v=\sqrt{\mu }/\sigma$$

(3) C=μv

This model tells only part of the story, a part that is relevant to a fast-paced environment where individuals do not “space-out” for long. If there are any microstates of inattention in this model (Cheyne et al., 2006), they will manifest as slow trajectories of the regular Wald distribution. Frank lapses of attention are treated in the next section. But now it is reasonable to try some worked examples.

### Application of the NeT

The SART is a popular measure of sustained attention (Smilek et al., 2010), with the originating article (Robertson et al., 1997) being cited over 600 times, often by other users of the procedure. The SART reverses the more common GO/NOGO vigilance procedure by requiring repeated responding (key presses) to a series of digits, and withholding responding when one of the 9 digits, typically “3”, appears. Subjects are usually told to respond as quickly as possible while maintaining high accuracy.

It is rare for researchers to report intra-subject variability, which is necessary to engage NeT. Of those that do, many fail to also report mean response times, or do it in normalized form; or substitute *p*-values for the data. One study that reported both first and second moments of the response distributions (Braet et al., 2009) was focused on the analysis of fMRI images, but provided statistics for the SART for 20 young adults and 20 young adolescents. The use of Eq. (2) permits us to recode arbitrary statistics into the variables of interest (assigning values for σ = *cv*μ, where *cv* is the reported coefficient of variation).

Table 1 shows that the inferred speed of computation, *v*, was greater for adults, a common finding. The criterion *C* was also larger for adults, reflecting less impulsivity in making a response. This moved their distribution to the right. If it took 440 ms for both groups to abort a response (*T*_stop_), then the inferred percentage of false positives is close to that measured by these investigators.

**Table 1:** **Application of Eq. (2) to the data of Braet et al. (2009)**.

	μ **(ms)**	**Coef. Var**.	**FP (%)**	***C***	***v***	**FP (% pred.)**
**GROUP**						
Adults	550	0.19	12	123	0.223	14
Adolescents	520	0.26	32	88	0.169	30

Such results were replicated by Carriere et al. (2010), who collected similar data on individuals ranging in age from 14 to 77. Through the lens of NeT, their data showed that available energy for responding increased to a peak in the fourth decade, and decreased thereafter; *C* increased monotonically with age.

**Figure 2:**
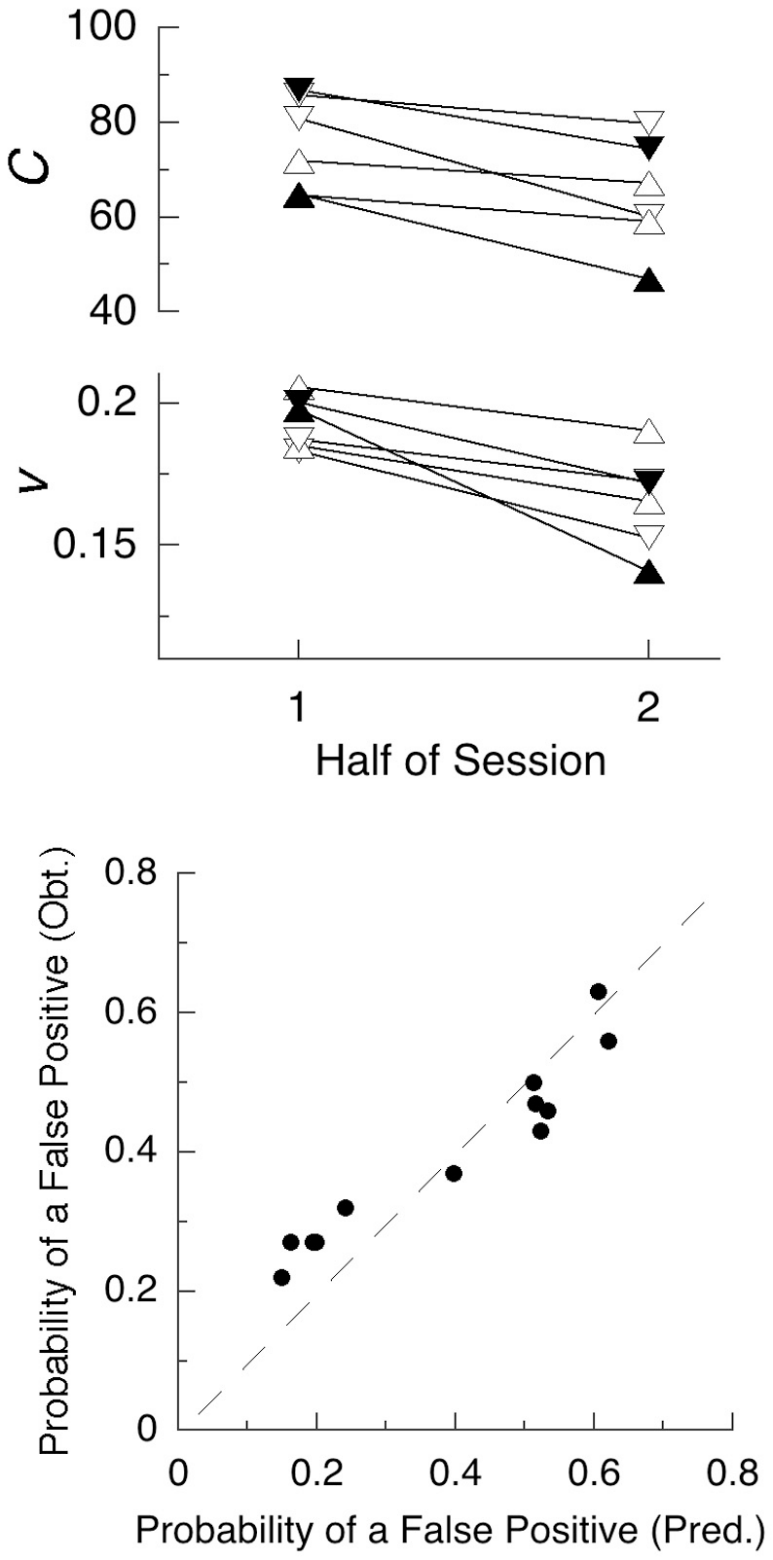
**Top Panel:** Summary statistics from two experiments using the SART (Seli et al., 2012a) entail these values for criterion (*C*) and speed of computation (*v*). Fatigue of functional units, here involving the brain circuits used for discrimination of the symbol “3”, is manifest by lower values of *v* in the second half of all experiments. The triangles denote standard instructions, and the inverted triangles “go-slow” instructions. Filled symbols are from the conditions with distracting audio alerts. **Bottom panel:** Assuming a value of *T*_stop_ = 343 ms predicts the probability of failing to abort a response on the no-go trials, as shown on the *x*-axis; the *y*-axis gives the obtained probabilities.

This study demonstrates the deployment of NeT, illustrating how it may be used to extract variables of interest from summary statistics. But a better exercise of the model is found in the data reported by Seli et al. (2012a). They used a standard SART design, but reported data from the first and second half of the session. In the first study, they had standard (equal emphasis on speed and accuracy) instructions, and a second group instructed to go slowly to improve accuracy (within the constraints of fast-paced trials). A second study replicated the first, and added periodic alerts intended to call the subjects to attention to the task (reminding them to “try and be very aware of what you are doing in the task.”). All conditions employed 30 college students in each. NeT predicts a decrease in energy available, and thus speed of computation, going from the first to second half, and increased criteria for the “go-slow” group (down triangles). Figure 2 shows the values imputed to these indices, which sustain the predictions.

Figure 2 also shows that there was a decrease in the criterion in the second half of the trials. If these were self-paced trials, the criteria may have remained constant, or increased to permit the same levels of accuracy in first and second halves. But, for both standard and “go-slow” instructions, the subjects had only 1 s in which to respond before the next stimulus appeared. The decreased criteria in the second half, when processing speeds were slower, may have been a strategy to avoid errors of omission, which were rare. Other investigators using the SART (e.g., McVay and Kane, 2009) also report a degradation in performance as a function of time through task, validating that basic prediction of NeT.

By increasing their criteria for a response, subjects in the “go-slow” conditions succeeded in reducing the false-positive responses: All six of the data points in the lower part of the lower panel come from that condition (one of those points lies hidden behind another in this graph). Assuming that it required *T*_stop_ = 343 ms to abort a response, we predict that the proportion of erroneous “go” responses shown on the *x*-axis of that panel that would have slipped through before they could be countermanded. This analysis requires that forced slowing of responding must reduce errors in this task, which has been demonstrated (Seli et al., 2012b).

### Attention loss and recapture

**Figure 3:**
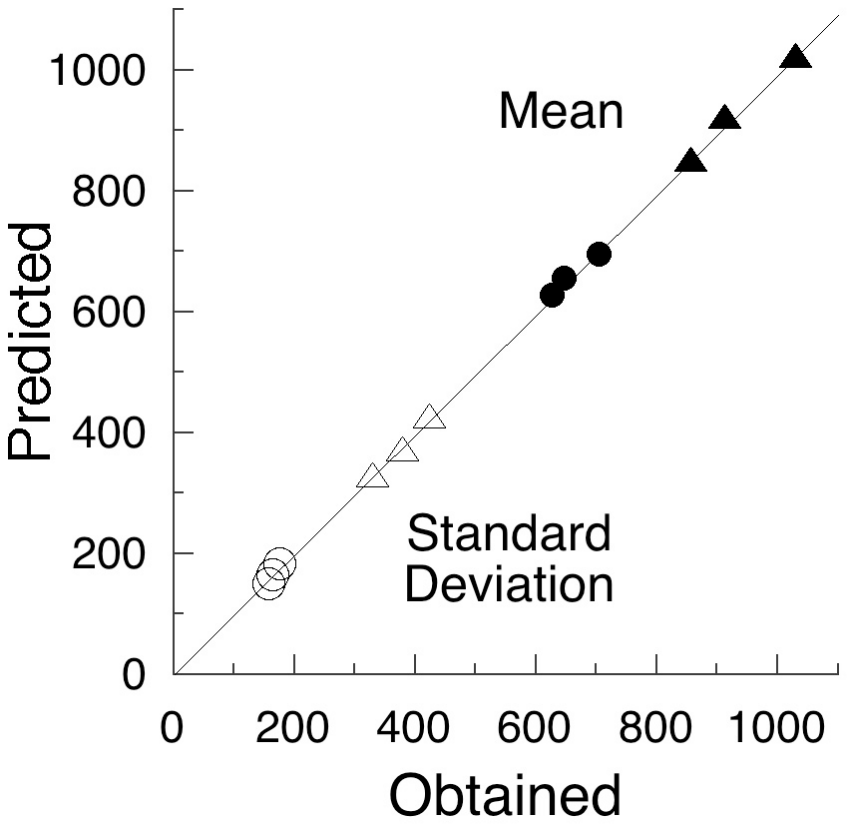
**The mean and standard deviations from a slow choice task (Leth-Steensen et al., 2000) are arrayed on the *x*-axis**. Children with ADHD are shown as triangles, controls as circles; filled symbols are means, unfilled standard deviations. Within clusters, the longer fore-periods had monotonically increasing values. The *y*-axis gives the values extracted from the model shown in Figure 4, using the parameters given in Table 2.

There is nothing in the above analysis that suggests lapses of attention. At most, one sees slowing speed of neural computations as a function of time on task, reflected in decreases in *v*; and changes in the criterial number of computations, indexed by *C*, as a function of task difficulty, temporal constraint, instructions, speed/accuracy tradeoffs, and population (i.e., age, DSM category, etc). The SART is too fast paced to abide gross lapses of attention and mind wandering. But a more slowly-paced, more boring task might do so. Such an experiment was reported by Leth-Steensen et al. (2000), who analyzed data from a study that presented four empty circles on a computer screen, initiating a fore-period of 2, 4, or 8 s. At the end of that wait, one of the circles was colored-in, and the participant had to press of one of four corresponding keys. There followed an inter-trial interval of 2.5 s, and then the next set of empty circles. The experiment lasted up to 3 h. The results from two of the groups analyzed, 17 boys with ADHD and 18 age-matched controls, are presented on the *x*-axis of Figure 3.

**Figure 4:**
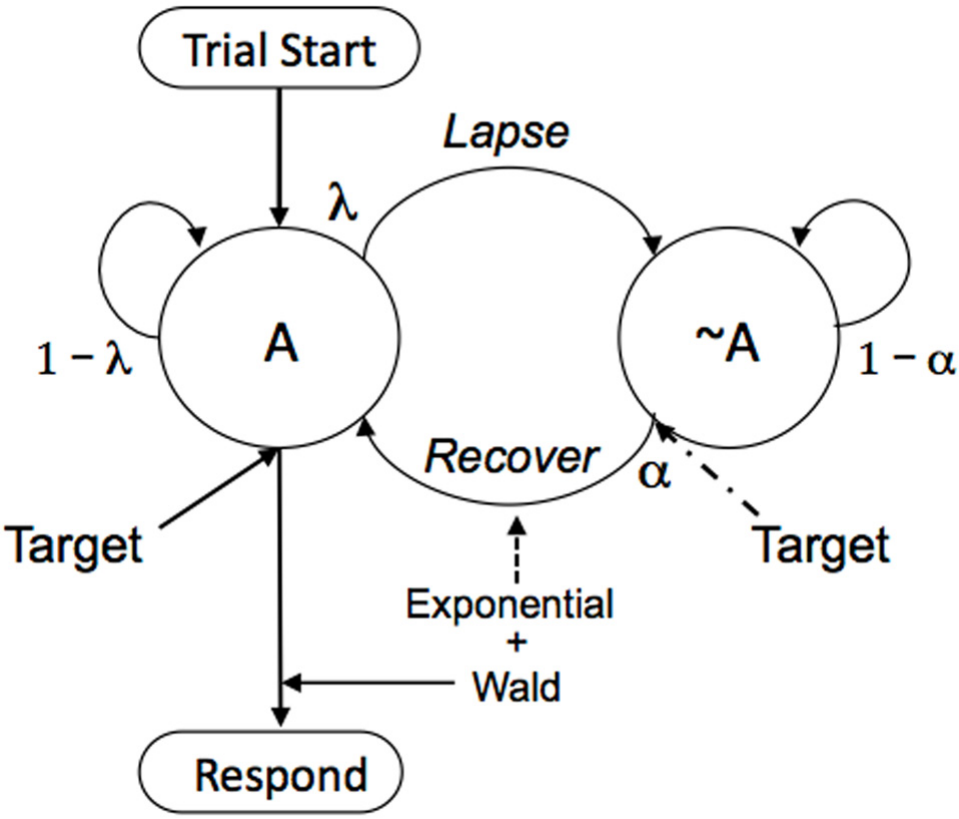
**Trials start the participant in the attentive (A) state; responses triggered by a target when in that state ensue according to Eq. (1)**. But there is a probability λ of attentional lapses from 1 s to the next, moving the participant to the inattentive state (~A). Responses triggered by a target from that inattentive state return attention to the task with probability α, and responses then ensue according to Eq. (1). If there is no target presentation, as is often the case in mind wandering and for non-experimental settings, attention will drift back slowly, with probability α much lower than when stimulated to do so by a conspicuous target.

There was a marked increase in mean and variance of response times as the waiting period increased. Although Eq. (1) could be fit to those data, it does not make sense for NeT to do that, as there is no principled reason for expecting radical slowing and increasing variance of neural processing over these relatively short intervals, which occurred in randomized blocks through the session. Therefore the model was expanded to include the separate process of attentional lapses, as shown in Figure 4.

This Neuroenergetics of Maintained Attention (NEMA) model is usefully simplified for two different contexts: Stimulus-driven recapture and goal-driven recapture. In the first case, involving experimental procedures with conspicuous stimuli, set the probability of recovery of attention (α; alpha) to 0 until a target presentation, and thereafter to a value close to 1. This results in a “double” exponential process: an exponentially increasing probability of lapsing attention as the trial progresses; and then an exponentially distributed delay to recapture. This is the case used for analysis of laboratory paradigms in general. The case of goal driven attention, the more general scenario for absent-mindedness and for detection of inconspicuous stimuli, is treated in Section 3.4.

The predictions for this model are those for the Wald, with additional time and variance added due to the probabilistic lapse of attention and its recovery:

(4) μ=C/v+(1−p(A))τ

(5) $$\sigma^{2}=C/v^{3}+(1-p(A))\tau^{2}$$

The probability of being in the attentional state decreases as *p*(*A*) = (1 − λ)^*t*^, where *t* is time through trial in seconds. τ (tau) is the mean of the exponentially distributed return time corresponding to α. The resulting distributions are a mixture of Wald and ex-Wald (Schwarz, 2001), with first two moments as given above. An implication of this model is that mind wandering will increase as the pace of the task decreases, and as its duration increases. Another is that frequent sojourns through the inattentive state will greatly increase variance in performance (Eq. 5), which has been shown to be the case (Seli et al., 2013).

With this attentional model, and the parameters given in Table 2, the predictions for the data reported by Leth-Steensen et al. (2000) are arrayed on the *y*- axis of Figure 3. The imputed probability of inattention increased from around 22% in the 2 s conditions to around 60% in the 8 s conditions.

**Table 2:** **Parameters for the data from Leth-Steensen et al. (2000)**.

	***C***	***v***	λ	τ **(s)**
**GROUP**				
ADHD	79	0.104	0.108	0.43
Controls	115	0.194	0.091	0.19

Whereas the accurate prediction of 12 data-points using a model with 8 parameters may not impress, the model is principled, and the parameter values throw light on the populations studied. The ADHD group had substantially less energy available (*v*), as expected, and thus their speeds of computation *v* were much less than for controls (see Table 2). Controls were able to make substantially more computations, as evidenced by the values of *C*. The probabilities of attentional lapse (λ; lambda) were about the same, but it took the ADHD group much longer to return to attention (τ).

When speed of processing is compromised, as in ADHD, this seriously impacts working memory, which requires quick processing to operate on information while maintain the availability of other information. Conversely, individuals with limited working memory capacity (WMC) are more likely to mind-wander (Kane and McVay, 2012). NeT claims that both limitations on WMC and mind wandering are caused by limitations of energetic resupply to focal neural groups.

### A mind adrift

Successful present-mindedness entails sensitivity to stimuli that guide behavior at choice-points. Who among us has never left the house only to have to return to it for keys, or eyeglasses, or phone, or lunch, or papers? A common algorithm to cope with such absent-mindedness is to set the item, or a sign of it, near the risky choice points. “If you cannot remember,” you can hear your mother saying, “set reminders”! Memoranda and to-do lists do not improve our memory, but rather obviate the need for it. A lazier and less-successful tactic is to make a mental note: “when I get to the back door I must check to be sure that I have my books”. There is a growing literature on “prospective memory,” remembering to do something at some point in time or in the presence of some stimulus, such as leaving for home at 3:30 today, and picking up some milk when driving past the grocery. Failure of the cues to elicit the uncommon temporal or physical detour indicates stronger control of behavioral trajectories by habits than by intentions. If you are interested in knowing more about prospective memory, do not make a point of remembering to check it out—Google-Scholar it now, and drag the URL to your desktop; your mother would be proud.

The practice of meditation gives one immediate witness to one's own inability to hold attention steadfast. A meditator attempts to keep his mind empty; or filled only with one object—breathing, or a mantra, or an object of devotion. This is impossible to maintain for long, as the mind goes delinquent almost immediately. This is not a problem for successful meditators, as their art is to bring it bring it back on topic; again and again and again. And with practice, the increasing periods of success are deeply quieting and gratifying.

Figure 4 provides a model of this process; but now there are no extrinsic targets being presented to refocus attention (or they are brief or inconspicuous). In the case of the meditator, the A state is correct focus on intended image, and the ~A state is attention to other ideas. In the case of the absent minded-professor, the A state is attention to, and sensitivity to, the cues at choice-points in the world around him; the ~A state is control by the momentum of habit, or by internal dialogs that must share attention with getting out the door. Upon starting meditation, the meditator is in the A state; and again there on returning a vagrant mind to the object of attention. Upon being charged with his task, the professor is in the A state; he makes a mental note to pick up the laundry list on the way out the door. But as time passes, with probability λ from 1 s to the next, attention will go vagrant. A different idea will occur to the meditator; the doorknob will loose its ability to cue the unfamiliar action to the professor. Then with probability α the meditator's attention will drift back to the object of veneration; and the professor will underscore his mental note.

To know where the mind will be at any point in time, we operate the Markov model shown in Figure 4. This is accomplished by first writing its transition matrix:

(6) $$P=\left|\begin{array}{cc} 1-\lambda & \lambda \\ \alpha & 1-\alpha \end{array}\right|$$

The top row of *P* corresponds to being in A and the bottom row ~A, at the current time. The first column corresponds to A at the next time-step, and the second column corresponds to ~A at the next time step. Thus, if currently in A, the top row, the individual will stay in A with probability 1–λ; but he will lapse to ~A with probability λ. If in ~A, he will recover attention with probability α, and remain absent minded with probability 1–α. To compute the presence or absence of mindedness after 1 time step, raise the matrix to the power 1, and inspect the top left cell. That is what you see before you—the exercise just completed. To compute the state after 2 time-steps, square the matrix; and after *n* time steps, raise it to the *n*th power. After enough time, no matter in which state the individual starts, the probability of being present-minded—of staying on task, or being sensitive to prospective memory cues that have been set—converges to the value α/(α + λ). If starting from the A state, there is a constant probability λ of loosing attention that, over many trials, sees attention winking out according to a concave function that resembles an exponential decay, falling to a floor of α/(α + λ). If starting from the absent-minded state ~A, there is a complementary increase, to a ceiling of α/(α + λ).

**Figure 5:**
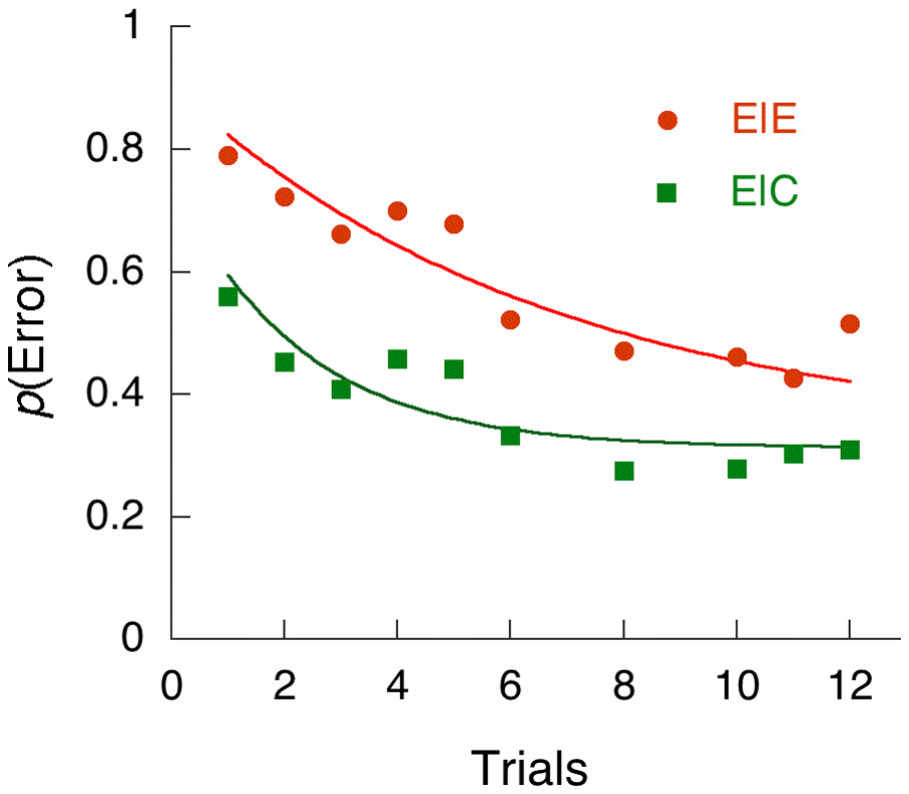
**The probability of an error (a positive response on a NOGO trial) after a prior NOGO trial**. The abscissae are trials since last NOGO trial. The ordinates are the probability of making an error of commission, conditional on whether the prior NOGO trial had occasioned an error (E|E), or a correct withholding of the response (E|C). The data are from Cheyne et al. (2009). The curves are from NEMA, under the assumption that a NOGO trial immediately throws the subject into the ~A state in order to process the rare event, from which they return according to the parameters of Table 3.

Sometimes the very act of perceiving a rare stimulus on a rapidly-paced task such as the SART will drive the subject into an error-processing mode, during which time attention to subsequent stimuli is affected. An illustration of this is provided by the research of Cheyne et al. (2009) who conducted a SART task with a large number of subjects, permitting the tracing of errors as a function of the number of trials since the previous rare event (a NOGO trial in the SART), when correctly (E|C) or incorrectly (E|E) responded to on that prior trial. These data are shown in Figure 5, along with the traces from Eq. (6). In order to account for these data within the framework of NEMA, I had to assume that the NOGO trial immediately sent the observer into an inattentive processing mode (~A) with greatly reduced sensitivity to stimuli (i.e., blind to them). Individuals then returned to attention from one trial to the next with the probabilities a given by Table 3. Figure 5 shows the data, and the model results from NEMA. In both cases, the curves converged on the same asymptote, 32% probability of an error.

**Table 3:** **Parameters for the data from Cheyne et al. (2009)**.

	α	λ
**CONDITION**		
Prior correct (E|C)	0.115	0.258
Prior error (E|E)	0.046	0.095

What this analysis shows is that, after a correct response on the rare NOGO trial, participants were much quicker to return to attention (α = 0.12) than after errors, on which they brooded three times as long (α = 0.046), and which caused an inevitable subsequent omission (see the Authors' Figure 7). But after an error, participants were much slower to lapse into an inattentive state (λ = 0.095) than after a correct inhibition. They were chastened by their error. Presence in the inattentive state will both slow responses and increase the probability of an omission of a go response, a correlation shown by these authors' Figure 4. The authors of this study called these effects *reactive mind wandering*, but perhaps a better name for it would be error processing.

## Summary

The parameters α and λ tell us the strength of attractor States A and ~A. To the extent that the former is larger than the latter, the individual will remain attentive. Of course, against the cause of A is the fact that there is seldom just one inattentive state; there are many byways that beckon the mind off its highway, each with fresh resources of energy to beguile. To maintain presence in state A (as in vigilance tasks) or sensitivity to cues which will return it to A (as in performance or prospective memory tasks) depletes energetic resources. According to the neuroenergetics theory NeT, many of these resources come from the astroglia that provide lactate, either by relatively direct conversion of glucose to lactate, or through the exploitation of stores of glycogen that astrocytes contain. As energy stores deplete, the ability to stay in A, indexed by 1–λ, depletes with it, and lapses will therefore increase with time on task. Processing of task-irrelevant stimuli is, of course, better when attention is not so highly focused on task (Weissman et al., 2009), and in some contexts this has survival value (Killeen et al., 2012). Whether mind wandering is considered a feature or a bug depends on context and outcome (McVay and Kane, 2010).

There are many threats to present-mindedness. Strong attractors may compete with attention to task, whether those are puzzling through a recondite scientific, logical or historic problem; or are worries over finances, health, or relationships. Conversely, attention may wander simply for lack of energy to maintain it in State A. Poor sleep and general fatigue are notorious as threats to attention, as deleterious to the maintenance of skilled performance as is alcohol. It is during sleep, especially slow-wave sleep, that the brain restores the astrocytes' supplies of glycogen (Benington and Heller, 1995). Without that “bench”, the neurons quick depletion of energy cannot receive adequate resupply from the astrocytes' slower processing of capillary-derived glucose.

Stimulants such as amphetamine and methylphenidate help maintain focus because, among other effects, they increase the presence of noradrenaline, and they do so preferentially in areas of the brain that are busy processing information, such as maintaining working memory for intellectual tasks. In doing that, they decrease the attractiveness of the fully energized other regions of the brain, and their ability to subvert attention. Noradrenaline stimulates the adrenoceptors of the astroglia, signaling them to convert glycogen to lactate, which is then shuttled to the neurons. When this system is derailed in any way, it takes focused attention with it. When brain glucose utilization was analyzed using Positron Emission Tomography, methylphenidate decreased glucose utilization in the parts of the brain that are associated with mind-wandering (Volkow et al., 2008), consistent with the NeT. One of the techniques used to maintain attention in individuals with ADHD is fidgeting. That activity increases sympathetic tone, and may facilitate noradrenergic release of lactate from astrocytes. It is interesting that it has recently been shown that such fidgeting is predicted by measures of inattention and spontaneous mind-wandering (Carriere et al., 2013).

Performance tasks such as the SART may deteriorate with time on task because of such fatigue, without mediation by gross failures of attention. As seen in Figure 5, however, errors may cause attention to be shifted to error correcting ruminations, causing increased probability of subsequent error. On slower-paced continuous performance tasks, attention will wander without the goad of error processing. Figure 4 and Eqs. (4–6) provide a model of that process. Although a relatively simple model, to adequately test its applicability to absent-mindedness will require confrontation with detailed data sets from slowly paced tasks.

One evening as a graduate student, the physicist I. I. Rabbi contemplated the enormous amount of tedious work he would have to do to over the next months to complete his dissertation project. Rather than start work, his mind wandered into fantasy and paths of whimsy. By the end of the night he had sketched a new analysis, and in just a few months had completed many times the research required for his Ph.D. He called the approach he fantasized and then invented “magnetic resonance” (Rigden, 1987). Mind wandering, as in that case, is often a feature, rather than bug: Fantasy is escapist, “and that is its glory. If a soldier is imprisoned by the enemy, do not we consider it his duty to escape?… If we value the freedom of mind and soul, if we are partisans of liberty, then it is our plain duty to escape, and to take as many people with us as we can!” (Tolkien, 1945). Perhaps not always though, especially if suturing an aneurism or piloting a 737.

### Conflict of interest statement

The author declares that the research was conducted in the absence of any commercial or financial relationships that could be construed as a potential conflict of interest.
